# Sonic Influence on Initially Neutral Brands: Using EEG to Unveil the Secrets of Audio Evaluative Conditioning

**DOI:** 10.3390/brainsci13101393

**Published:** 2023-09-29

**Authors:** Shannon Bosshard, Peter Walla

**Affiliations:** 1 School of Psychology, Newcastle University, University Drive, Callaghan, NSW 2308, Australia; shannon.bosshard@uon.edu.au; 2 Faculty of Psychology, Freud CanBeLab, Sigmund Freud University, Sigmund Freud Platz 1, 1020 Vienna, Austria; 3 Faculty of Medicine, Sigmund Freud University, Sigmund Freud Platz 3, 1020 Vienna, Austria

**Keywords:** neutral brands, evaluative conditioning (EC), electroencephalography (EEG), event-related potentials (ERPs), neuromarketing, information technology, NeuroIS, neuroconsulting

## Abstract

The present study addresses the question of whether explicit, survey-type measures of attitude differ in sensitivity when compared to implicit, non-conscious measures of attitude in the context of attitude changes in response to evaluative conditioning (EC). In the frame of a pre-test, participants rated 300 brand names on a Likert-type scale, the results of which were then used to create personalised lists of neutral brands. After this initial online component, the participants were exposed to one, five, and ten rounds of EC (during three separate sessions), during which half of the brands were paired with pleasant audio excerpts (positive EC) and the remainder were paired with unpleasant audio excerpts (negative EC). Following each conditioning round, the participants rated the brand names again, whilst changes in the brain’s electrical activity in response to the brands were recorded via electroencephalography (EEG). After having rated the brand names, the participants also completed two implicit association tests (IAT; one for each of the neutral conditions). The results revealed that self-reported, explicit responses of brand names remained unchanged despite having been conditioned. Similarly, the IAT did not reveal any declines in reaction time. In contrast, the EEG data appeared to not only be sensitive to initial brand ratings, but also the conditioning effects of initially neutral brands. Respective neurophysiological effects were found at frontal electrode locations AF3 and AF4 for a 1 s-long time window starting at 400 ms after stimulus onset. Furthermore, the EEG revealed that changes in brand attitude are more susceptible to the effects of negative conditioning than positive conditioning. Given the rather small sample size, any generalizability seems vague, but the present results provide scientific evidence that EEG could indeed be a valuable additional method to investigate EC effects. The results of this study support the notion of utilising a multidimensional approach, inclusive of neuroscience, to understanding consumer attitudes instead of solely relying on self-report measures. In the end, the brain knows more than it admits to consciousness and language, which is why objective methods should always be included in any study.

## 1. Introduction

Attitudes serve as a critical differentiator between stimuli that elicit appeal and those that provoke avoidance. Gawronski and Galen [1] underscore the significant role of attitudes in shaping attention allocation and stimulus interpretation. In the context of the contemporary competitive advertising landscape, brand differentiation stands as a requisite strategy. Amidst endeavors by marketing agencies to reshape consumer perceptions of their offerings, only a fraction of brands manage to attain recognition, favorability, and consistent purchase behaviour. Given the limited exploration of neutral brands within the existing literature, our present study seeks to investigate the impact of evaluative conditioning on neutral brands through the utilisation of both explicit and implicit measures.

Irrespective of the limited number of research papers seen to investigate neutral brands, it is well-established that neutral brands exist. Both the marketing and the psychology literature acknowledge that well-established brands that are liked and disliked are associated with strong positive or negative feelings, respectively [2]. References made to both well-liked and disliked brands imply that neutral brands must exist. With a greater understanding of the attitudes towards brands comes an increased ability to modify consumers’ attitudes towards them. In cases where a brand is perceived as neutral, successful marketing is essential.

Although advertising may initially seem straightforward, the underlying mechanisms that drive changes in attitudes remain largely unknown. And whilst advertisers and marketers have developed numerous strategies and tactics to influence consumer attitudes and behaviour, the exact processes and variables that lead to these changes can be intricate and not always fully understood. Considerable research within the field of psychology has revealed numerous theories that help provide an understanding of the relationship between consumer attitudes and brands. Arguably, the most valuable insights have come from the conditioning literature. Conditioning is the process by which behaviours and responses are learned through associations formed between stimuli and outcomes, leading to changes in individual attitudes, preferences, and actions. Interestingly though, within branding contexts, the conditioning literature has revealed that well-established brand attitudes are often resistant to the effects of advertising [3,4,5,6].

One point of contention, regarding the conditioning literature, or the psychology literature more generally, is the notion that consumers either cannot or do not want to fully explain their preferences [7,8]. These findings have been shown to occur across both commercial and academic domains, challenging our assumptions about how preferences are formed and communicated. As these findings become more prominent, they cast a revealing light on the complexities of human decision-making processes. The implications of such insights extend far beyond theoretical considerations; they bear practical significance for businesses and researchers alike.

Despite the challenges of understanding consumer preferences persisting, it is worth noting that advancements in technology have led to more sophisticated, sensitive technologies capable of detecting such changes. The field of neuroscience has established that there are at least two types of attitudes. Explicit attitudes are said to be contemplative and formulated through reasoning [1]. This type of attitude is assessed using traditional, self-report measures (e.g., surveys and focus groups). As consumers undergo the process of reasoning at a conscious level, higher-order structures are called upon and this inevitably produces a negative effect referred to as cognitive pollution [9]. Cognitive pollution clouds the judgement of the consumer, and when questioned about their attitude towards a product or brand, the consumer, although able to provide a response, has provided a contaminated insight into their attitude.

In contrast, implicit attitudes are associations that are automatically activated in the presence of relevant stimuli without any conscious awareness of evaluation [10]. Implicit attitudes have repeatedly been shown to contradict explicitly stated responses. That is, negative associations can be activated even if the individual subjectively perceives their outlook towards it to be positive, and vice versa [11]. Furthermore, implicit attitudes are shown to be considerably robust [12] and better predictors of spontaneous behaviour [13]. As a result, the authors of the current paper propose that gauging a thorough understanding of consumer attitudes can only come from simultaneously using a combination of implicit and explicit attitudes.

Of the tools used to investigate implicit attitudes, the implicit association test (IAT; see Greenwald et al.) [14] is the most commonly cited. The IAT is a reaction time-based task and has been utilised within psychological research to measure non-conscious attitudes in relation to social prejudices including racism and stereotypes [8,14,15,16]. The IAT is arguably the most popular and effective response-latency-based implicit measure, even within consumer contexts. It has been met with a number of criticisms, however, regarding legitimacy as a reliable and valid index of implicit attitudes [17,18,19,20].

An emerging alternative to the IAT is electroencephalography (EEG). This measure of non-conscious processing has been shown as a useful technique for obtaining implicit information. But, despite its usefulness, EEG’s adoption within attitude research and, more specifically, consumer/commercial attitude research is limited [21]. Of the available research, EEG has been shown to be sensitive to positive (approach) and negative (avoidance) effects [22], in addition to changes in attitude [23] and implicit processes in general [24,25]. Typically, greater relative left frontal EEG activity is said to be associated with the processing of positive/approach effects, whilst greater relative right frontal EEG activity is said to be associated with the processing of negative/avoidance effects [22]. Within applied contexts, Ohme [26] reported increases in approach-related behaviour whilst participants viewed marketing-related information (TV commercials; seen as increases in left frontal activity) whilst product–benefit, product, and brand scenes were presented. Furthermore, Handy et al. [27] found that when participants rated logos as positive, these stimuli elicited more activity than those that were rated negatively. Of most interest for the present study, the most empirically valid EEG approach as an index of motivation and effect has been a distinct event-related potential (ERP) component, the late positive potential (LPP). The LPP has been extensively used within the literature and, as a result, has received psychometric endorsement [28]. Stimuli that are seen to be either more emotionally affective or more motivationally significant are said to evoke the largest LPPs (Moran et al., 2013). These pieces of literature also suggest that greater LPP activity is usually generated over right hemisphere electrode sites during evaluative tasks [29].

The present study extends on the study by Walla et al. [9] in that it utilises EEG and IAT as implicit measures of effect. They [9] investigated brand attitudes but focused on startle reflex, heart rate, and skin conductance. This study, to our knowledge, is one of only two of its kind and is unique in that it allowed the participants to view well-established brands that they were familiar with over subsequent conditioning sessions. The participants were initially presented with a lengthy list of well-known brand names and explicit attitudes were collected to be used as baseline measures. Following this collection, the participants visited the laboratory on numerous occasions where subsequent sessions aimed at modifying attitudes to initially neutral brands took place. We hypothesised that implicit and explicit measures would be differently sensitive to the effects of evaluative conditioning of initially neutral brands, and thus, their combined use will provide a more thorough understanding of brand attitude and attitude changes.

## 2. Materials and Methods

### 2.1. Participants

The current study enlisted the participation of 22 individuals, from whom 2 were subsequently excluded based on a preliminary assessment of brand attitudes. The remaining group comprised 20 participants (10 females) with a mean age of 22.81 (SD = 2.37). All participants were university students enrolled at the University of Newcastle, Australia. Their involvement was voluntary and predicated upon written informed consent. The participants were eligible to participate if they were right-handed, had normal or corrected-to-normal vision, were free of medication or substances impacting the central nervous system (including alcohol, caffeine, and nicotine), and had no history of neuropathology (see Table 1). Compensation for time and travel was provided to the participants. Ethical approval for the study was granted by the Newcastle University Ethics Committee (H-2013-0038).

### 2.2. Stimuli

The initial set of stimuli utilised for pre-assessment consisted of 300 commonly recognised brand names, selected subjectively to reflect brands familiar to individuals in Australia. Through an online survey, the participants were tasked with assigning a subjective rating of either liking or disliking to each brand name. This rating was conducted on a 21-point analog scale, encompassing a range from −10 (strong dislike) to 10 (strong like). Concurrently, brand names that garnered a neutral rating of zero from the participants constituted the compilation of neutral brands. The final list for each participant consisted of 60 neutral brand names and 80 non-target (filler) brand names. The neutral brands were further categorised into two distinct groups: Condition N (neutral brands conditioned negatively) and Condition P (neutral brands conditioned positively).

#### Conditioning Stimuli

For the purpose of conditioning the target (neutral) brand names, auditory stimuli sourced from the International Affective Digitized Sound System (IADS) were employed, see [23]. This collection comprises 111 emotionally charged sounds, each having undergone comprehensive pre-evaluation in terms of emotional valence (and arousal). This standardisation facilitates the matching of sounds based on affective attributes (either positive or negative). From these 111 sounds, 30 unpleasant sounds were selected (the mean pre-evaluated valence rating was 3.1; SD = 0.57), whilst another 30 pleasant sounds were selected (mean valence rating of 6.7; SD = 0.75) and used to condition the neutral brands. The mean pre-evaluated arousal ratings for both categories were standardised at 6.6 (with only slight variations in their SDs—0.48 for negative sounds and 0.41 for positive sounds). Notably, all sound stimuli were randomly paired with each of the brand names as part of the evaluative conditioning process.

### 2.3. Procedure

#### 2.3.1. Individual Pre-Assessment of Brand Attitudes

Prior to their lab sessions, the participants took part in an online survey hosted on https://www.limesurvey.com to subjectively rate 300 brand names. Using a mouse or trackpad, the participants employed a slider interface to express their attitudes toward each brand name. To indicate a neutral attitude, the participants were instructed to position the slider at the midpoint labeled “0”. If a brand name was unfamiliar, the participants were advised to abstain from interacting with the slider for that specific item. The initial survey was completed at the participants’ convenience, using their personal computers. On average, the survey took approximately 15–20 min to finish. To proceed to the experimental phase of the study, the participants needed to demonstrate both adequate familiarity with the brands and a diverse range of attitude responses. Notably, two participants were excluded from further participation due to inadequate results from the brand pre-assessment phase.

#### 2.3.2. Lab Experiment

Participants meeting the eligibility criteria were subsequently invited to return to the laboratory within a few days of completing the initial brand assessment. In this initial session, baseline measurements of both explicit and implicit attitudes toward the brand names were gathered. Self-report measures were employed to capture explicit, subjective responses, while the collection of implicit, non-conscious responses involved the use of electroencephalography (EEG) and the implicit association test (IAT). The presentation of stimuli to the participants occurred on a 32” LED television with a resolution of 1024 × 768 pixels. To record brain potential changes, a 64-electrode BioSemiActiveTwo EEG system (BioSemi, Amsterdam, The Netherlands) was utilised. Additionally, eight external reference electrodes were positioned laterally around the eyes, above and below the eyes, and on the mastoids.

The software program “Presentation (16.4)”, (NeuroBehavioral Systems in Albany, USA), was employed for visually presenting the instructions, customized stimulus lists, and positive/negative sounds sourced from the International Affective Digitized Sounds (IADS). All physiological recordings and monitoring of participants took place in a separate room. Despite the participants’ anticipated familiarity with the experiment, a recap of the procedure was provided during the setup phase. Once the setup was finalised, the participants initiated testing individually in a dimly lit room to ensure optimal concentration on the presented stimuli.

**Figure 1:**
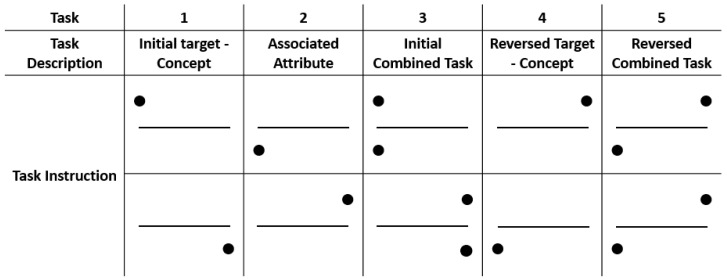
Modified version of the original IAT (adapted from Greenwald et al. [14]). Filled black circles on the left or right of the stimulus correspond to left and right button presses (respectively). Task 3 = congruent, Task 5 = Incongruent condition. The participants completed this task during each of the lab sessions, once for each neutral condition.

Each brand stimulus appeared as a white text against a black background for a duration of 5 s, interspersed with a white fixation cross displayed for 500 milliseconds between each presentation. The participants were instructed to rate their attitude toward each brand using a standard keyboard, providing ratings on a scale ranging from 1 (strong dislike) to 9 (strong like). Data collection encompassed both brain potential changes and self-reported responses for both the set of 60 target brands and 80 filler brands. A brief mid-stage break was incorporated to mitigate the potential effects of participant fatigue. On average, this entire phase of the study was completed in approximately 30 min. Following this break, the participants engaged in five rounds of the implicit association test (IAT) (please refer to Figure 1 for the modified IAT design).

After completing the implicit association test (IAT), the participants were required to complete several rounds of conditioning: one round in session 1, five rounds in session 2, and ten rounds in session 3, totaling 16 rounds. Each conditioning round lasted approximately six minutes, and the participants had the option to take breaks as necessary. Upon the completion of the IAT, the participants scheduled their next session before leaving the lab. The time span between lab visits was standardised to the best of our ability, with the participants required to attend subsequent sessions within a range of two to five days from their previous session.

### 2.4. Data Recording and Processing

#### 2.4.1. Explicit Data

Paired-sample *t*-tests were conducted to compare mean self-reported ratings. Among the participants’ personalised brand lists, 60 neutral brands were categorised into 30 for negative conditioning (Condition N) and 30 for positive conditioning (Condition P). Self-report ratings collected prior to conditioning for both Condition N and Condition P were merged and used as a baseline measure.

#### 2.4.2. Implicit Association Test (IAT)

This study utilised a modified version of the original implicit association test [14]. This adapted IAT included five distinct discrimination tasks, each comprising 30 visual presentations classified as either target or non-target stimuli (see Figure 1). From the pool of 60 brands previously rated as neutral by the participants, 30 underwent negative conditioning (Neutral Condition N), while the remaining 30 underwent positive conditioning (Neutral Condition P). These 60 brands became the target brands. In the initial session, responses toward the neutral brands were aggregated and used as a baseline condition referred to as ‘Combined Session 1’.

In task 1 (initial target concept), the participants in the study were tasked with distinguishing between visual stimuli that were either connected to their individually rated neutral brands (target brand) or their most liked (or disliked) brands (non-target brand). The participants were required to press the “A” key for the target brand and the “L” key for the non-target brand. Throughout task 2 (associated attribute), the participants were presented with valenced words and instructed to press the “A” key for pleasant words (e.g., beautiful, healthy, happy, and perfect) and the “L” key for unpleasant words (e.g., frightened, angry, sad, and worthless). Task 3 (initial combined task) combined tasks 1 and 2 to form the congruent condition. The participants were directed to press the “A” key for the target brand and pleasant words and the “L” key when presented with a negative word or a non-target brand. Task 4 (reversed target concept) resembled task 1 but with the participants instructed to press the “A” key for non-target brands and the “L” key for target brands. Finally, task 5 (reversed combined task; incongruent condition) blended elements from task 2 and task 4. The participants were required to press the “A” key for non-target brands and pleasant words and the “L” key when presented with a negative word or a non-target brand. A comparative analysis was performed between the reaction times of the participants during task 3 (congruent condition) and task 5 (incongruent condition).

During each block, stimuli were presented for a duration of 300 ms, although the participants were provided 1500 ms to respond during each trial. A 300 ms fixation cross was displayed between each stimulus, followed by an additional 700 ms gap between the fixation cross and the subsequent stimulus.

#### 2.4.3. Event-Related Potentials

EEG recordings were captured at a sampling rate of 2048 samples per second, utilising a 64-channel BioSemiActiveTwo system paired with ActiView software; 8.09 (BioSemi, Amsterdam, The Netherlands). Individual data sets were processed using EEG-Display (version 6.3.13; Fulham, Newcastle, Australia). During processing, the sampling rate was downscaled to 256 samples per second, and a bandpass filter of 0.1 Hz to 30 Hz was applied. To rectify blink artifacts, referencing was applied to the supraocular external electrode (excluding two sets referenced to Fpz due to signal irregularities). Mitigation of noise attributed to eye movement was carried out via horizontal, vertical, and radial eye movement corrections [30].

The data were categorised by brand type (Neutral Condition N, Neutral Condition P, and Filler), and epochs were defined from −100 ms before stimulus onset (serving as the baseline) to 2000 ms after stimulus onset. After baseline correction of the resultant epochs, averages were computed across single trials for each condition. Subsequently, individual data sets were re-referenced to a mastoid reference electrode, and grand-averaged event-related potentials (ERPs) were generated to visualise variations in brain activity across the participants.

The initial EEG recording was averaged across Neutral Conditions N and P, later used as a baseline to ascertain subsequent conditioning effects. For statistical analysis, the epochs were segmented into 200 ms blocks, and mean amplitudes were statistically analysed via Analysis of Variance (ANOVA). Following the review of the main effects, randomization tests [31,32] were executed to assess differences between the conditions at various time frames.

Given that numerous studies have centered on frontal and parietal sites in exploring attitudes and behaviour [33,34,35,36], our study also focused on similar sites. Specifically, we chose frontal electrode sites AF3 and AF4, alongside parietal sites P5 and P6.

## 3. Results

### 3.1. Self Report

**Figure 2:**
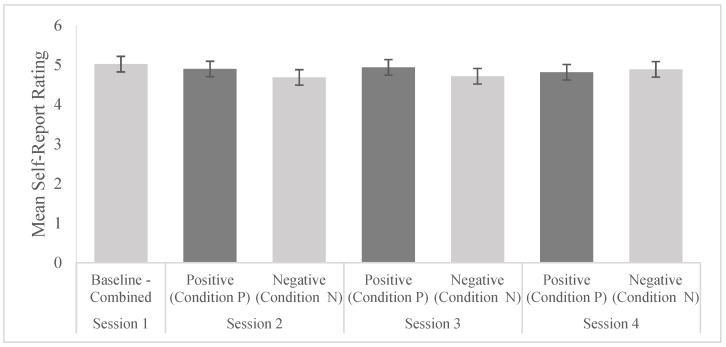
Mean self-report ratings of both neutral conditions (N and P) across all four sessions. The bars represent error bars.

We conducted a one-way ANOVA to compare the responses across all four sessions for both neutral conditions. An average across both conditions for session 1 was taken as no conditioning had been undertaken. Regardless of the conditioning direction (F(1.679) = 0.874, *p* = 0.350) or session (F(2.679) = 0.074, *p* = 0.929), no significant differences in explicit rating were seen for either condition. Figure 2 shows a respective bar diagram.

### 3.2. IAT

**Figure 3:**
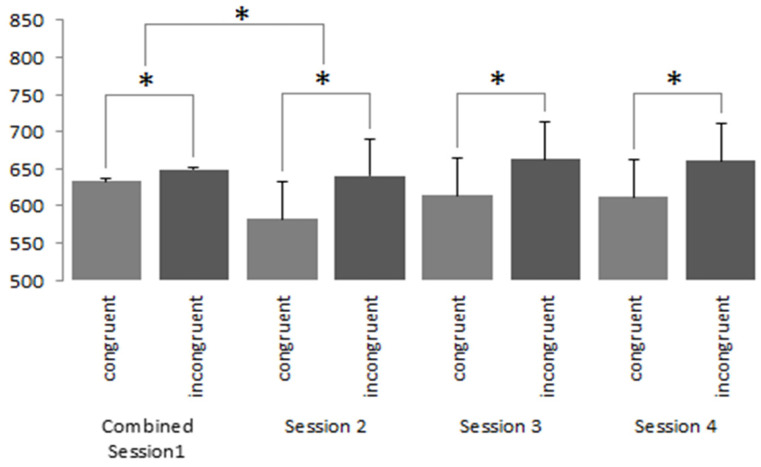
IAT results (in milliseconds) reflecting responses towards brands that were conditioned negatively (Neutral Condition N) in the congruent and non-congruent conditions. * denotes a *p*-value less than 0.001. The bars represent single-sided error bars.

During analysis of the IAT responses, we began by removing all responses that fell three standard deviations from the overall mean of each phase. We also removed all incorrect responses and then analysed the data pertaining to the participants’ neutral brands. Neutral brands conditioned negatively revealed a significant effect of session (F(3.690) = 9.84, *p* < 0.001, ηp^2^ = 0.041). Subsequent post hoc tests using Bonferroni corrections revealed a significant (*p* < 0.001) decline in reaction time between session one and session two for both the congruent (M = 631.31, SD = 148.60; M = 582.29, SD = 997.82) and incongruent conditions (M = 656.81, SD = 136.10; M = 639.67, SD = 123.08). However, the remaining sessions saw an increase in reaction time. A main effect of condition was seen (F(1.230) = 584.33, *p* < 0.001, ηp^2^ = 0.718), which saw congruent conditions elicit faster reaction times than incongruent conditions (M = 109.17, SD = 130.24; M = 654.54, 148.13; *p* < 0.001; see Figure 3).

**Figure 4:**
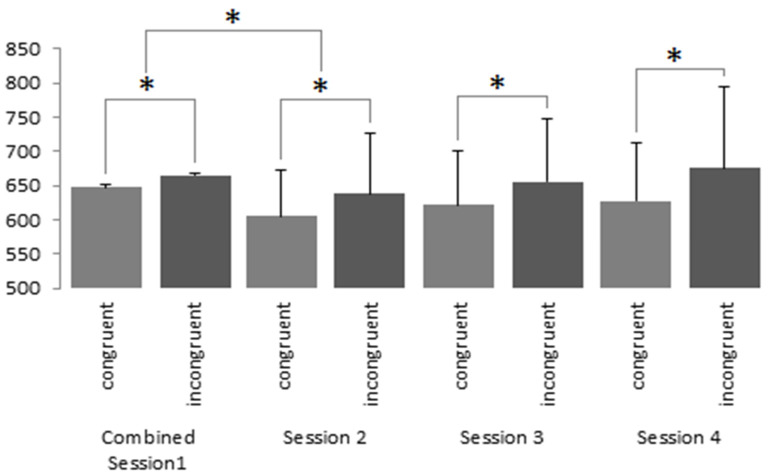
IAT results (in milliseconds) reflecting responses towards brands that were conditioned positively (Neutral Condition P) in the congruent and non-congruent conditions. * denotes a *p*-value less than 0.001. The bars represent single-sided error bars.

Similarly, brands conditioned positively saw a significant effect of session (F(3.690) = 7.191, *p* < 0.001, ηp^2^ = 0.030). Subsequent post hoc tests using Bonferroni corrections revealed no significant declines (*p* = 0.069) in reaction time between session one and session two for either the congruent (M = 633.35, SD = 151.23; M = 608.36, SD = 106.18) or incongruent conditions (M = 655.68, SD = 139.78; M = 638.33, SD = 133.74). Again, no further declines in reaction time were seen throughout subsequent sessions. A main effect of condition was seen (F(1.230) = 265.73, *p* < 0.001, ηp^2^ = 0.536), which saw congruent conditions elicit faster reaction times than incongruent conditions (M = 623.21, SD = 128.63; M = 568.56, 153.19; *p* < 0.001; see Figure 4).

### 3.3. Event-Related Potentials

Firstly, for frontal sites AF3 and AF4, a 2 (hemisphere: left, right) × 2 (conditioning valence: positive, negative) × 4 (session: one, two, three, four) repeated measures ANOVA was conducted for each 200 ms block between 400 ms and 1800 ms. From 400 ms through to 1000 ms, a significant main effect of the session was present and was most significant at approximately 700 ms (F(3.57) = 6.399, *p* = 0.001, ηp^2^ = 0.252). Also present was a significant interaction effect of conditioning valence and session between 800 ms and 1400 ms. This effect was seen to achieve the greatest significance at approximately 1000 ms (F(3.57) = 3.865, *p* = 0.014, ηp^2^ = 0.169). In contrast, no significant results were reported with regards to a hemisphere by session nor a hemisphere by valence interaction, ruling out any frontal asymmetry effects.

Similarly, a 2 (hemisphere: left, right) × 2 (conditioning valence: positive, negative) × 4 (session: one, two, three, four) repeated measures ANOVA was conducted for each 200 ms block between 400 ms and 1800 ms for parietal sites P5 and P6. A significant main effect of hemisphere was reported from 800 ms and remained for the duration of the epoch (F(1.19) = 30.707, *p* < 0.001, ηp^2^ = 0.618). Pairwise comparisons saw that the right electrode site P6 elicited significantly greater positive activity than its left counterpart, electrode P5.

#### 3.3.1. Brands Conditioned Negatively

Firstly, a 2 (hemisphere: right, left) × 4 (conditioning rounds: zero, one, five, ten) repeated measures ANOVA was conducted. Initial results across frontal sites (AF3 and AF4) revealed significant effects of session (F(3.57) = 4.408, *p* = 0.007, ηp^2^ = 0.188) but not hemisphere. To further investigate session effects, ERPs were processed in 200 ms blocks, beginning at 400 ms. ANOVAs were conducted for each 200 ms block and revealed session effects between 400 ms (F(3.57) = 3.965, *p* = 0.012, ηp^2^ = 0.173) and approximately 1400 ms (F(3.57) = 3.681, *p* = 0.017, ηp^2^ = 0.162), with greatest significance occurring at approximately 900 ms (F(3.57) = 5.455, *p* = 0.002, ηp^2^ = 0.233).

**Figure 5:**
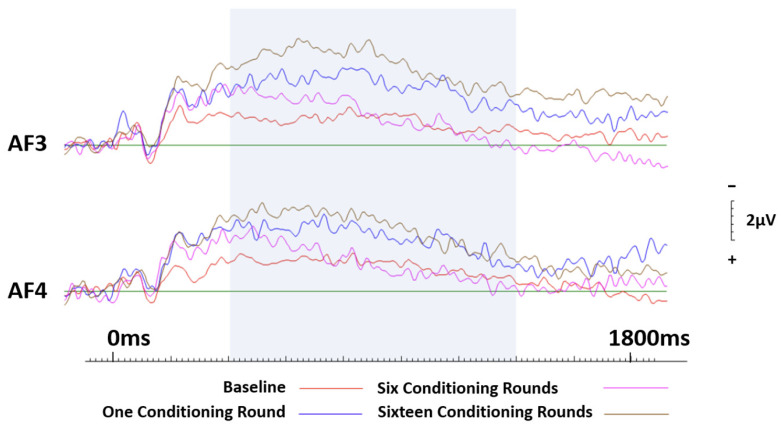
ERP curves generated at baseline and again after one, six and sixteen conditioning rounds for neutral brands that were conditioned negatively. Frontal sites AF3 and AF4 only. The highlighted section indicates the area of most significance.

To confirm these findings, further analysis saw the implementation of a Monte Carlo permutation-based analysis (randomization test) [31,32]. Randomisation tests are considered to be an equally conservative (nonparametric) approach when compared to traditional methods; however, there is no reliance on having normalised data. Randomised correlations were conducted in the same manner above (every 200 ms), to assess the relationship between individual sessions. Using this approach, any instances where correlations are significant, present support for the null hypothesis (i.e., no difference in session). For left frontal site AF3, between 400 ms and 600 ms, a significant correlation was evident (r = 0.65, *p* = 0.001, two-tailed); however, no additional significant correlations between baseline recordings and subsequent sessions were reported. In contrast, across frontal site AF4, significant correlations were witnessed between baseline measures and sessions 2 and 3 (r = 0.612, *p* = 0.005, two-tailed), indicating similarities in the recordings. Only between the baseline and session 4 were no significant correlations present, indicating that the two sessions were in fact different. See Figure 5.

**Figure 6:**
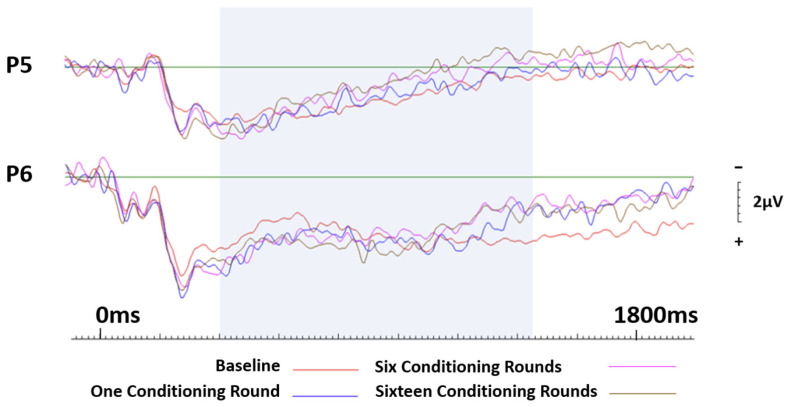
ERP curves generated at baseline and again after one, six, and sixteen conditioning rounds for neutral brands that were conditioned negatively. Parietal site P5 and P6 only.

Across parietal sites, no session effects were recorded for neutral brands conditioned negatively. However, significant lateralisation effects were witnessed, with the right electrode site, P6, eliciting significantly greater activity than the left electrode site, P5 (F(1.19) = 18.289, *p* < 0.001, ηp^2^ = 0.490). See Figure 6.

#### 3.3.2. Brands Conditioned Positively

**Figure 7:**
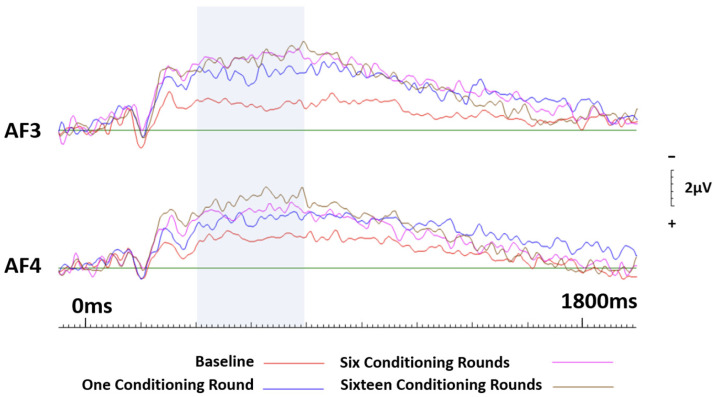
ERP curves generated at baseline and again after one, six and sixteen conditioning rounds for neutral brands that were conditioned positively. Frontal sites AF3 and AF4 only. The highlighted section indicates the area of most significance.

A 2 (hemisphere: right, left) × 4 (conditioning rounds: zero, one, five, ten) repeated measures ANOVA was conducted. The initial results across the frontal sites (AF3 and AF4) revealed no significant effects of session (F(3.57) = 1.760, *p* = 0.165, ηp^2^ = 0.085), or hemisphere (F(1.19) = 1.485, *p* = 0.238, ηp^2^ = 0.072). Despite the non-significant findings pertaining to the session, visual inspection of the ERPs demonstrates a considerable difference between the baseline and all subsequent sessions. For this reason, ANOVAs were conducted for each 200 ms block in a similar manner described above. The results revealed a significant effect of the session between 400 ms (F(3.57) = 6.148, *p* = 0.001, ηp^2^ = 0.244) and approximately 800 ms (F(3.57) = 6.770, *p* = 0.001, ηp^2^ = 0.263; see Figure 7). Randomised correlations, as described above, were conducted to further assess session effects, with a primary focus on differences between baseline and subsequent conditioning sessions. Non-significant correlations were indicative of differences between sessions. With this in mind, results showed that only the baseline and session four (16 conditioning rounds) were not correlated for the entire duration of the epoch. In contrast, the baseline and sessions two and three were both significantly correlated (r = 0.60, *p* = 0.003, two-tailed) from 400 ms until approximately 1200 ms.

**Figure 8:**
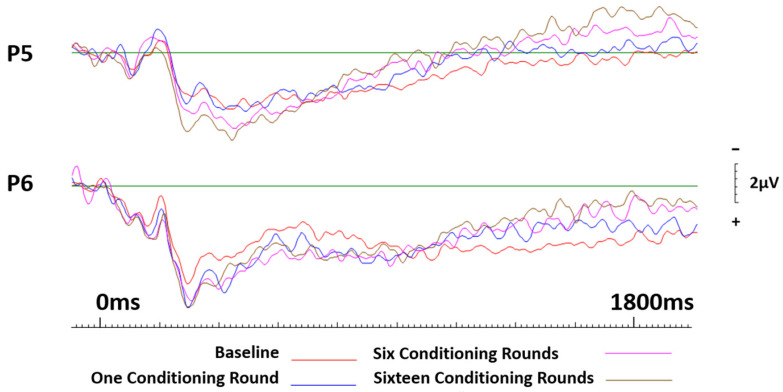
ERP curves generated at baseline and again after one, six, and sixteen conditioning rounds for neutral brands that were conditioned positively. Parietal sites P5 and P6 only.

Across the parietal sites, no session effects were recorded for neutral brands conditioned positively; however, lateralisation effects revealed that the right electrode site, P6, elicited significantly greater activity than the left electrode site, P5 (F(1.19) = 22.366, *p* < 0.001, ηp^2^ = 0.541). See Figure 8.

#### 3.3.3. Filler Brands

Following a similar approach to the neutral conditions (N and P), we conducted repeated measures ANOVAs in a 2 (hemisphere: left, right) × 2 (conditioning valence: positive, negative) × 4 (session: one, two, three, four) design for each 200 ms block spanning the 1400 ms epoch. This analysis focused on filler brands at frontal and parietal sites. No significant main effects or interactions emerged for filler brands at either frontal (AF3 and AF4) or parietal sites (P5 and P6). It is important to note that the filler brands were presented consistently without conditioning, mirroring the treatment of the target brands. No discernible session effects were observed across the frontal sites [23]. Although there are slight gradual increases in negativity across sessions, these effects lack statistical significance, thereby negating the influence of brand name repetition effects when examining target brand conditioning (see Bosshard et al. for an in-depth analysis of filler brands [23]).

## 4. Discussion

Using a personalised approach to conditioning, we utilised individual brand lists, based on participants’ self-reported brand ratings, to investigate changes in attitudes for neutral brands. Subsequent visits to the lab, which saw the participants undergo one, six, and sixteen rounds of evaluative conditioning via brand/audio pairings, saw self-reported attitudes towards the initially neutral brand remain unchanged. Similarly, the IAT did not reveal any declines in reaction times as a result of conditioning, and thus, very few conclusions can be drawn. In contrast, EEG appears to be the only tool sensitive to the effects of evaluative conditioning of initially neutral brands. The filler brands further substantiate EEG as a useful tool to both assess initial brand attitudes, and the effects of evaluative conditioning. Together, these findings reiterate those reported in previous publications, that self-report, the IAT, and EEG are differently sensitive to attitudes and also subsequent changes in said attitudes.

### 4.1. Self Report and the IAT

Although the results collected during the pre-assessment phase indicated that the participants were able to differentiate between liked, disliked, and neutral brands [23], we found no differences in explicit ratings over subsequent sessions following evaluative conditioning. This supports the notion that components of brand attitude are located deep within subcortical structures (or in the non-conscious mind) and are not accessible by conscious processes. Whilst these findings are unusual, they are not surprising. Previous research seen to investigate attitudes, particularly within the field of psychology, suggests that latent inhibition may explain the lack of findings [37,38,39]. Latent inhibition describes the inability of an individual to learn new information about a stimulus with which they have had previous exposure. However, within marketing and consumer contexts, it has been well established that advertising campaigns can change attitudes and, thus, consumer-based behaviour. A more likely explanation of the null findings, founded upon neuroscience research, is that of cognitive pollution, proposed by Walla, Brenner, and Koller [9]. Cognitive pollution is the process whereby an explicit response becomes polluted as a result of the conscious evaluation of a stimulus [9,40]. This explanation is in line with much of the existing literature, which suggests that up to 95% of all consumer behaviour is driven by processes that occur outside of an individual’s awareness [41] and that there are two streams of attitude [42], one that is accessible via conscious processes (i.e., explicit) and one that occurs outside of conscious awareness (i.e., implicit).

The results regarding the IAT suggest that it may be sensitive to changes in attitudes; however, these findings should be interpreted with caution. Although it is clearly the case that the IAT is able to differentiate between positive and negative affect (congruent and incongruent conditions), it does not seem to be sensitive to conditioning effects. While significant effects between the first and second sessions were found, throughout the subsequent sessions, the reaction times were seen to increase. The lack of findings reiterates those reported in previous papers, that the IAT may not exclusively measure implicit attitudes, but instead, be influenced by cognition. For instance, De Houwer [17] and Gregg et al. [43] reported that merely instructing partcipants to imagine that one of the groups with which they were presented was positive (good, peaceful, etc.) and that the second was negative (bad, violent, etc.), resulted in participants responding more quickly to the compatible/congruent condition than the incompatible/incongruent condition.

### 4.2. Event-Related Potentials

Of all the measures utilised within the current paper, EEG seemed to be the most sensitive to the effects of evaluative conditioning of neutral brands. These results reiterate the need for marketers to become less reliant on self-report measures. Within consumer contexts, motivation, be it approach or avoidance, plays an important role in an individual’s intentions to purchase or engage with a brand [44,45,46]. Our results affirm that the brands utilised within the current study did not elicit strong motivational responses. Late positive potentials are reported to be generated in the presence of affective stimuli across parietal regions [47]. Given the findings of previous studies, which suggest that LPP effects should be equally enhanced for both pleasant and unpleasant stimuli [28,29], it is no surprise that the neutral brands within the current study were processed similarly regardless of the direction of conditioning.

With regards to frontal asymmetry, it was expected that neutral brands that were conditioned positively would elicit greater activity across the left frontal sites [22]. Unexpectedly, our results revealed that neither frontal hemisphere showed dominance during the presentation of either of the neutral conditions. From this finding, it is inferred that the brands, although having been conditioned, were unable to elicit strong affective responses. These findings possibly arose given that brands, unlike the typical stimuli utilised within attitude research, are not as intrinsically emotionally arousing. Typically, attitude research is seen to focus on associations that are innate and stronger (e.g., out-group prejudices [48]). In contrast, as mentioned previously, brand attitudes are entirely learned and highly semantic [39]. However, this is not to say that further conditioning would not have resulted in effects.

In contrast, parietal sites saw greater activity across right electrode sites during the presentation of both neutral conditions. This finding can be explained using an asymmetry model presented by Heller [49] which is seen to extend upon that presented by Davidson et al. [22]. Although it supports the notion proposed by Davidson et al. [22] that frontal activation is determined by emotional valence, it goes further by suggesting that parietal regions are involved in the modulation of autonomic and behavioural arousal. Furthermore, Heller postulates that higher activation across the right parietal regions is associated with higher autonomic arousal. Given that both neutral conditions elicited lateralisation effects that saw greater right parietal activation during their presentation, it is therefore suggested that whilst conditioning did not evoke changes in valence towards the brands, the participants did find the brands arousing after conditioning.

Of all the findings related to our ERPs, those that are most interesting relate to evaluative conditioning effects. Although all three rounds of conditioning elicited changes in implicit attitudes, a single round of conditioning was enough to elicit changes in implicit processing, regardless of the direction of conditioning. This finding is in line with a substantial volume of literature that suggests that one round of conditioning will typically elicit the largest change in attitude, whereas subsequent conditioning trials will evoke only smaller changes until a maximum is reached [39,50,51]. This being said, more research is required before conclusions can be drawn given that the majority of the existing conditioning literature either presents null findings or uses fictitious brand stimuli.

The ERP findings presented within the current paper emphasise the importance of successful marketing strategies for neutral brands. Whilst one round of conditioning elicited the largest change in implicit attitudes for neutral brands conditioned positively, the effects of negative conditioning seem to be far longer lasting. Within the psychology and marketing literature, it has been proposed that a negativity bias exists, whereby negative stimuli generally acquire more attention than those that are positive [52,53,54,55]. The results presented in the current paper reiterate those presented in the extant literature and suggest that negative stimuli appear to be a more potent unconditioned stimulus. Within an applied setting, there is an overwhelming amount of evidence that links negativity bias with a decrease in consumer sentiment [56,57,58,59]. What is more surprising is that, whilst this reduction in sentiment has a negative effect on consumption, increases have little to no effect [52]. All in all, it is likely the case that positive associations within consumer contexts are simply of less importance to us and thus more difficult to establish [60].

### 4.3. Conclusions

In the present study, self-report, ERP measures, and the IAT were demonstrated to be sensitive to brand attitudes. However, only ERPs seemed to be sensitive to the effects of positive and negative evaluative conditioning of neutral brands. These findings affirm the fact that brands and, more specifically, brand attitudes are highly iterated and reprocessed constructs that are generally not well understood. The lack of any differences in LPPs and front hemisphere dominance revealed that the brands viewed by the participants were indeed neutral. In terms of conditioning, neutral brands that were paired with negative sounds revealed larger effects than neutral brands paired with positive stimuli.

It is essential that several recommendations for future research be made. It is imperative that a distinction be made between neutral brands with which participants are familiar, and those that are well-known, yet remain neutral. Having made this distinction clear, the findings presented in future papers will be relevant within applied settings. Authors within this field must acknowledge that implicit attitudes exist, and as a result, the current overreliance on fictitious brand stimuli is unnecessary. On that note, it is essential that future research utilises a combination of explicit and implicit measures of attitude. Traditional measures are plagued with inadequacies and allow for biases to arise in the data.

Like every other study, this investigation also has its limitations, which are mainly its small sample size (reasonable for an ERP study, but small from an objective perspective) and a related lack of generalizability as well as the fact that largely Australian brands were used (again not allowing generalizability). However, our findings are significant, and a final recommendation is aimed at marketers. Whether marketing a brand, product, or individual (e.g., political campaign), it is important to have an understanding of the implicit mind of the consumer. Although at a conscious level, attitudes may remain the same, negative events can have dire consequences on the implicit attitudes of an individual. When building strong, positive relationships, it appears to be the case that a great deal of work can come unstuck when an individual is simply exposed to just a single negative association. A very recent review of EC’s past, present, and future [61] did not mention a single brain imaging study. Our study confirms the usefulness of brain imaging, especially ERPs to investigate EC and to contribute to a better understanding of it.

## Figures and Tables

**Table 1:** Participant demographics (all healthy participants).

Demographics	n	Age	Education	Handedness	Vision
	20	22.81	University	Right-	Corrected
	(10 female)	(SD = 2.37)	students	handed	or normal

## Data Availability

Data are available on request.
